# RORα Regulates Odontoblastic Differentiation and Mediates the Pro-Odontogenic Effect of Melatonin on Dental Papilla Cells

**DOI:** 10.3390/molecules26041098

**Published:** 2021-02-19

**Authors:** Jun Kang, Haoling Chen, Fuping Zhang, Tong Yan, Wenguo Fan, Liulin Jiang, Hongwen He, Fang Huang

**Affiliations:** 1 Hospital of Stomatology, Guanghua School of Stomatology, Sun Yat-sen University, Guangzhou 510055, China; kangj5@mail2.sysu.edu.cn (J.K.); chenhl28@mail2.sysu.edu.cn (H.C.); zhangfp6@mail.sysu.edu.cn (F.Z.); yant6@mail2.sysu.edu.cn (T.Y.); fanweng@mail.sysu.edu.cn (W.F.); jliul@mail2.sysu.edu.cn (L.J.); 2 Guangdong Provincial Key Laboratory of Stomatology, Guangzhou 510080, China; 3 Guanghua School of Stomatology, Sun Yat-sen University, Guangzhou 510080, China

**Keywords:** nuclear receptor, retinoid acid receptor-related orphan receptor α, melatonin, dental papilla cells, odontoblastic differentiation

## Abstract

Dental papilla cells (DPCs), precursors of odontoblasts, are considered promising seed cells for tissue engineering. Emerging evidence suggests that melatonin promotes odontoblastic differentiation of DPCs and affects tooth development, although the precise mechanisms remain unknown. Retinoid acid receptor-related orphan receptor α (RORα) is a nuclear receptor for melatonin that plays a critical role in cell differentiation and embryonic development. This study aimed to explore the role of RORα in odontoblastic differentiation and determine whether melatonin exerts its pro-odontogenic effect via RORα. Herein, we observed that RORα was expressed in DPCs and was significantly increased during odontoblastic differentiation in vitro and in vivo. The overexpression of RORα upregulated the expression of odontogenic markers, alkaline phosphatase (ALP) activity and mineralized nodules formation (*p* < 0.05). In contrast, odontoblastic differentiation of DPCs was suppressed by RORα knockdown. Moreover, we found that melatonin elevated the expression of odontogenic markers, which was accompanied by the upregulation of RORα (*p* < 0.001). Utilising small interfering RNA, we further demonstrated that RORα inhibition attenuated melatonin-induced odontogenic gene expression, ALP activity and matrix mineralisation (*p* < 0.01). Collectively, these results provide the first evidence that RORα can promote odontoblastic differentiation of DPCs and mediate the pro-odontogenic effect of melatonin.

## 1. Introduction

Oral diseases such as dental caries, pulp and periodontal diseases, dental trauma and tooth loss are major public health problems worldwide, affecting the general health and quality of life of individuals [1]. Currently, conventional treatments are widely used in clinical practice, including root canal therapy, dental prostheses, and implants; however, these techniques cannot completely recover pulp vitality and tooth function. Therefore, the promising therapeutic strategy of regenerative medicine is gaining much attention for oral diseases because they can repair or regenerate various damaged dental tissues [2,3]. To facilitate the application of dental regeneration, it is essential to understand the process of tooth development. Dental papilla cells (DPCs), derived from the cranial neural crest, are precursors of odontoblasts and are responsible for dentinogenesis; thus, they play an indispensable role in tooth development [4,5,6]. With the induction of the inner enamel epithelium, undifferentiated DPCs first become preodontoblasts and then elongate and polarize, and finally differentiate into mature odontoblasts to synthesise and secrete predentin [7]. When the root is incompletely formed, dental papilla residing in the apical zone is called the apical papilla, which still contains a number of mesenchymal stem cells [8]. These cells contribute to root development, even in the regenerative endodontic procedures [9,10]. Furthermore, DPCs have the potential to self-renew and differentiate into functional odontoblast-like cells in vitro; therefore, they are considered promising seed cells for dental tissue regeneration [11,12,13]. However, the precise mechanisms underlying odontoblastic differentiation of DPCs are not fully understood. The exploration of related mechanisms will undoubtedly contribute to the development of dental tissue engineering.

Melatonin (N-acetyl-5-methoxytryptamine) is mainly synthesised in and secreted by the pineal gland in a circadian rhythm. It has diverse physiological and pharmacological functions, including free radical scavenging, antiaging, immune regulation and reproduction control [14,15,16,17,18]. These predominant effects are generally mediated by membrane receptors (MT1 and MT2), nuclear receptors, cytosolic binding sites (MT3 and calmodulin) and mitochondria [19,20,21]. Recently, numerous studies have shown that melatonin regulates the development of various tissues and organs, including the skin, hair, bone, liver and the nervous system [22,23,24,25]. In addition, melatonin has been extensively studied in the oral cavity, such as in bone remodelling, osteointegration of dental implants, periodontal disease and oral cancer; however, the effect of melatonin on tooth development and regeneration has not received sufficient attention [26,27,28,29,30]. Previously, we demonstrated that melatonin promoted the odontoblastic differentiation of DPCs in vitro and affected dentin formation in vivo, thus indicating that melatonin may play a critical role in tooth development [31,32]. However, the precise mechanisms underlying these effects have not yet been elucidated.

The retinoid acid receptor-related orphan receptors (RORs), which are members of the steroid hormone receptor superfamily, comprise three distinct subtypes, RORα, RORβ, and RORγ, which exhibit a typical structure containing four functional domains—a highly variable N-terminal domain, a highly conserved DNA-binding domain, a C-terminal ligand-binding domain and a hinge domain [33]. The molecular functions of RORs are usually achieved by regulating gene transcription through binding as a monomer to the ROR response elements (ROREs), comprising an AGGTCA motif preceded by an A/T-rich sequence in the promoter regions of target genes [34,35]. RORα is broadly expressed in various tissues and is considered a crucial regulator of many biological processes, such as circadian rhythm, metabolism, embryonic development and cellular differentiation [36,37,38,39]. In RORα-deficient mice, RORα has been shown to participate in the regulation of the development of the cerebellum, hair and lymphocyte [36,40,41]. Studies have also shown that RORα regulates adipogenic differentiation and myogenic differentiation [42,43]. Moreover, growing evidence suggests that RORα is involved in bone metabolism. In an in vitro experiment, RORα1 promoted the expression of osteogenic markers and inhibited TNF-induced NF-κB activation, which is critical for bone resorption [44,45,46]. In another in vivo study, RORα (−/−) mice exhibited thin long bones and osteopenia when compared to the heterozygote and wild-type animals, indicating that bone formation and maintenance were impaired [47]. These findings indicate that RORα is a positive regulator of bone development; however, no information is currently available regarding the functional role of RORα in odontoblastic differentiation and tooth development. In addition, RORα is known as the nuclear receptor for melatonin, and thus, it shares some effects of melatonin on oxidative stress, immune response, cardiovascular disease and liver fibrosis [48,49,50,51]. However, whether RORα mediates the pro-odontogenic effect of melatonin remains unknown.

Both dentin and bone are hard tissues, and their formation has many similarities. Moreover, in previous studies, we found that the pro-odontogenic effect of melatonin is not mediated by membrane receptors [31]. Based on this, we hypothesised that RORα could regulate odontoblastic differentiation and that melatonin promotes DPC differentiation in an RORα-dependent manner. To test this hypothesis, we first examined the expression pattern of RORα both in vitro and in vivo and then determined the role of RORα in the odontoblastic differentiation of DPCs by overexpression and knockdown of RORα. Finally, we investigated the influence of RORα on melatonin-induced odontoblastic differentiation. Our study would improve a deep understanding of tooth development and provide a potential target for dental tissue regeneration.

## 2. Results

### 2.1. Expression of Nuclear Receptor RORs in Rat DPCs (rDPCs)

**Figure 1:**
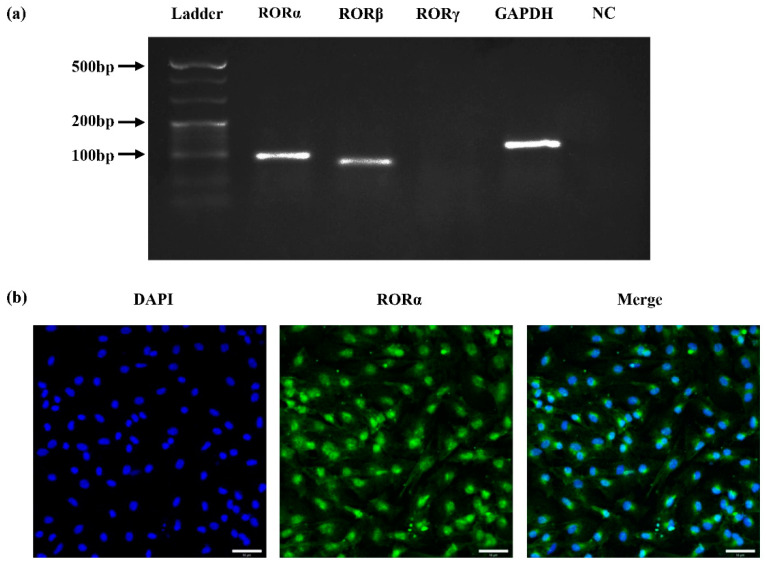
Expression of nuclear receptor retinoid acid receptor-related orphan receptors (RORs) in rat dental papilla cells (rDPCs). (**a**) Total RNA was extracted from rDPCs and detected by reverse transcription polymerase chain reaction (RT-PCR) and agarose gel electrophoresis assay using specific primers for RORα, RORβ, RORγ, and glyceraldehyde-3-phosphate dehydrogenase (GAPDH). RT-PCR without primers served as the negative control (NC); (**b**) Immunofluorescence staining was performed with polyclonal anti-RORα antibody (green), and nuclei were labelled with 4-6-diamidino-2-phenylindole (DAPI, blue). Scale bar: 50 μm.

To detect whether nuclear receptor RORs are expressed in rat DPCs (rDPCs), we performed reverse transcription polymerase chain reaction (RT-PCR) and agarose gel electrophoresis assay using specific primers for RORα, RORβ, and RORγ. RT-PCR data revealed that RORα and RORβ, but not RORγ, were expressed in rDPCs; moreover, the brightness of band of RORα was higher than that of RORβ (Figure 1a). Immunofluorescence staining further confirmed the presence of RORα at the protein level and showed that the RORα protein was mainly localised in the nuclei of rDPCs (Figure 1b).

### 2.2. RORα Is Upregulated during Odontoblastic Differentiation of rDPCs In Vitro

**Figure 2:**
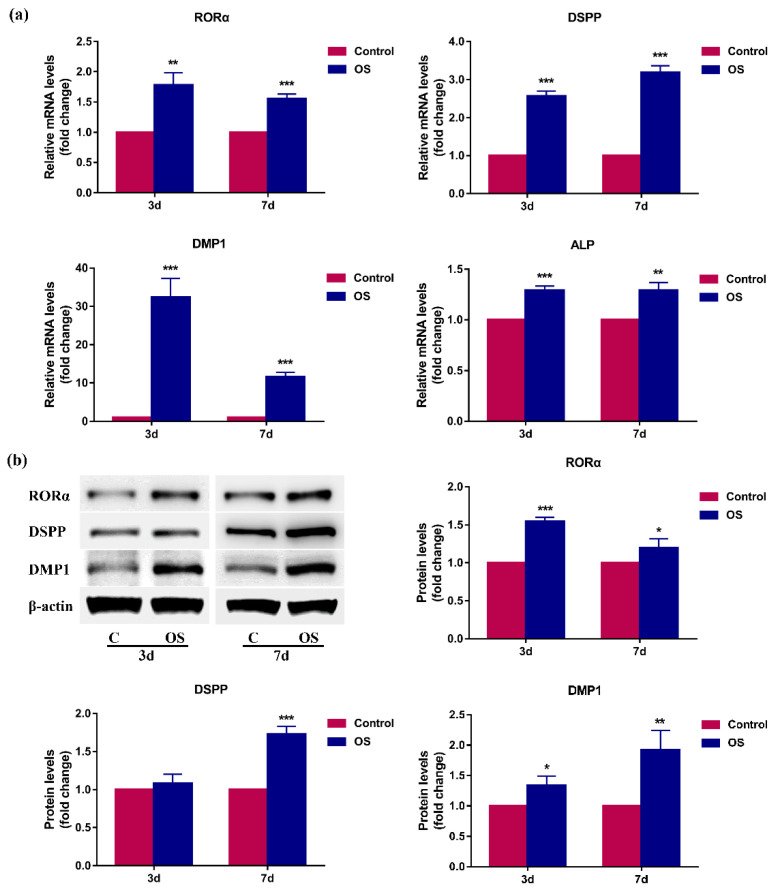
The expression pattern of retinoid acid receptor-related orphan receptor α (RORα) and odontogenic markers in rat dental papilla cells (rDPCs) during odontoblastic differentiation. rDPCs were cultured in maintained medium (control) or odontogenic induction medium (OS) for 3 and 7 days (3d and 7d), respectively. (**a**) The mRNA levels of RORα, dentin sialophosphoprotein (DSPP), dentin matrix protein 1 (DMP1), and alkaline phosphatase (ALP) were quantified by quantitative reverse transcription polymerase chain reaction (qRT-PCR). Glyceraldehyde-3-phosphate dehydrogenase (GAPDH) was used as the normalisation control; (**b**) The protein levels of RORα, DSPP, and DMP1 were determined by western blotting and normalised to the protein level of β-actin. All data are presented as the mean ± SD (*n* = 3). * *p* < 0.05, ** *p* < 0.01, *** *p* < 0.001 vs. Control group.

To explore whether RORα is involved in odontoblastic differentiation, we first determined the expression pattern of RORα in rDPCs during odontoblastic differentiation. rDPCs were cultured in an osteogenic/odontogenic induction medium (OS) for 3 or 7 days and then the mRNA and protein levels of RORα and odontoblastic markers were detected by quantitative RT-PCR (qRT-PCR) and western blotting, respectively. After 3- and 7-day induction, the mRNA levels of odontoblast-related genes, including dentin sialophosphoprotein (DSPP), dentin matrix protein 1 (DMP1), and alkaline phosphatase (ALP), were remarkably elevated (Figure 2a). Consistently, the protein level of DSPP was increased on day 7, and the protein level of DMP1 was upregulated on days 3 and 7 (Figure 2b). These data indicated that DPCs were successfully induced to differentiate into odontoblasts. Interestingly, RORα was significantly upregulated at the mRNA and protein levels following odontoblastic induction on both days 3 and 7 (*p* < 0.05), which implied that RORα may be involved in odontoblastic differentiation (Figure 2a,b).

### 2.3. RORα Is Increased during Odontoblastic Differentiation In Vivo

**Figure 3:**
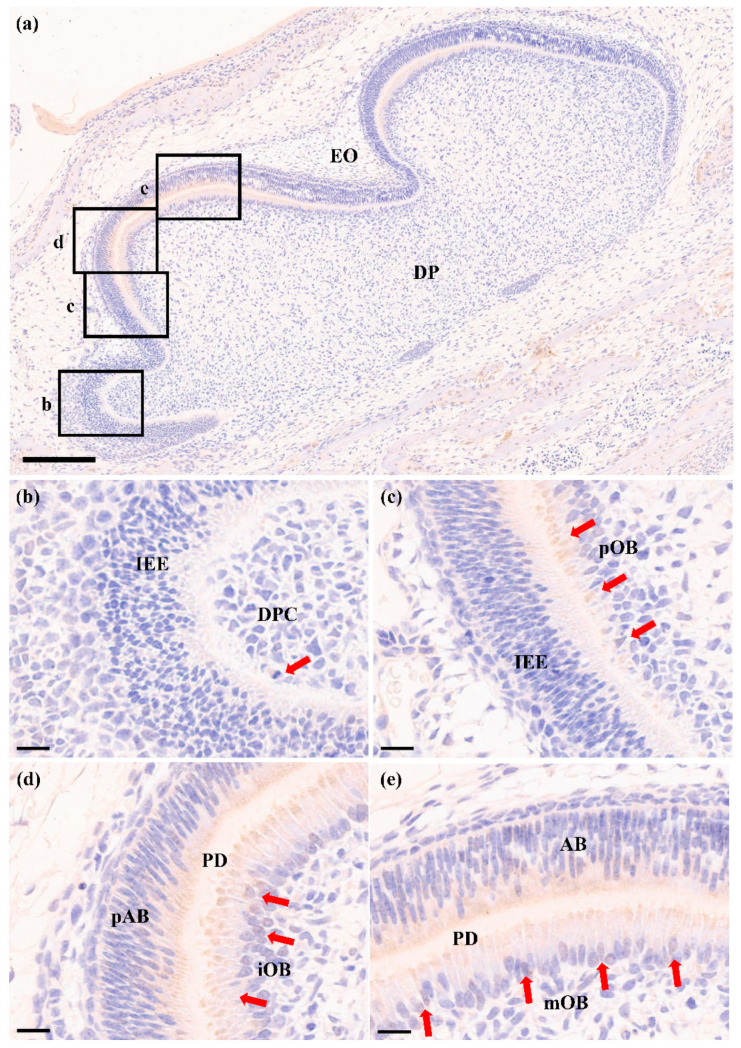
The expression pattern of retinoid acid receptor-related orphan receptor α (RORα) during odontoblastic differentiation in vivo. (**a**) Immunochemistry analysis showed the changes in expression levels of RORα during odontoblastic differentiation in the first lower molar of 1-day postnatal Sprague-Dawley rats; (**b**) RORα was weakly expressed in undifferentiated DPCs; (**c**) The expression of RORα began to increase in preodontoblasts; RORα was strongly expressed in immature odontoblasts (**d**), and the intense expression of RORα was maintained in mature odontoblasts (**e**). Red arrows mark RORα-positive cells. AB, ameloblasts; DP, dental papilla; DPC, dental papilla cells; EO, enamel organ; IEE, inner enamel epithelium; iOB, immature odontoblasts; mOB, mature odontoblasts; pAB, preameloblasts; PD, predentin; pOB, preodontoblasts. Scale bar: (**a**) 200 μm; (**b**–**e**) 20 μm.

To further verify that RORα was increased during odontoblastic differentiation, we detected the protein expression pattern of RORα during odontoblastic differentiation in the first lower molar of 1-day postnatal Sprague-Dawley rats. Immunochemical analysis showed all stages of the differentiation of DPCs into odontoblasts (Figure 3a). RORα was detected in undifferentiated DPCs; however, this expression was weak (Figure 3b). In preodontoblasts, the RORα expression levels began to increase (Figure 3c). With the progression of odontoblastic differentiation, RORα was strongly expressed in immature odontoblasts (Figure 3d), and the intense expression of RORα was maintained in mature odontoblasts (Figure 3e). These results suggested that RORα participated in odontoblastic differentiation and dentin formation. In addition, RORα was observed in preameloblasts and ameloblasts (Figure 3d,e).

### 2.4. Overexpression of RORα Promotes Odontoblastic Differentiation in rDPCs

**Figure 4:**
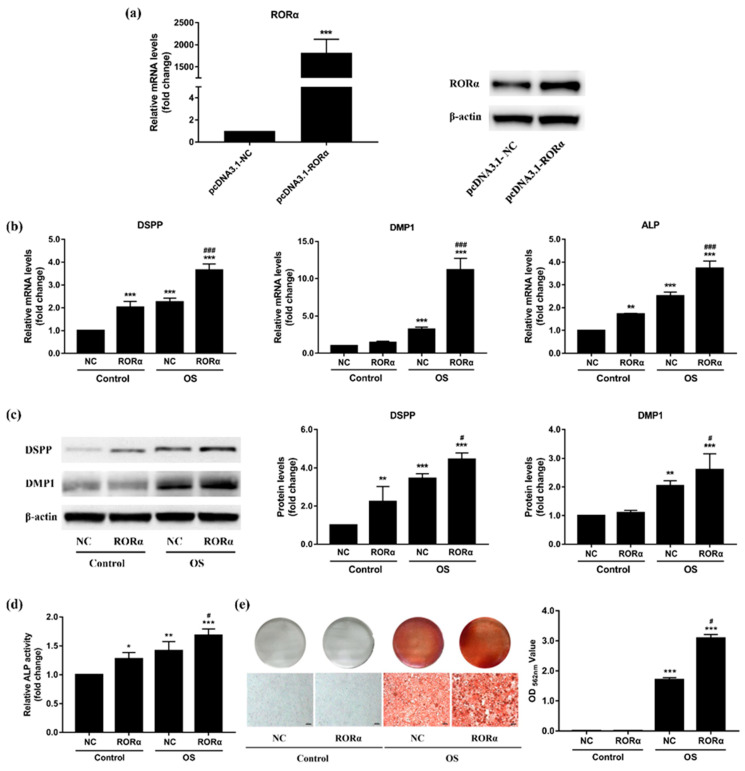
Effect of retinoid acid receptor-related orphan receptor α (RORα) overexpression on odontoblastic differentiation of rat dental papilla cells (rDPCs). rDPCs were transfected with pcDNA3.1-RORα (RORα group) or pcDNA3.1-NC (negative control group) for 24 h and then cultured in control or odontogenic induction (OS) medium for 3 or 7 days. (**a**) The transfection efficiency of RORα overexpression was assessed by quantitative reverse transcription polymerase chain reaction (qRT-PCR) and western blotting. (**b**) The mRNA levels of dentin sialophosphoprotein (DSPP), dentin matrix protein 1 (DMP1), and alkaline phosphatase (ALP) were detected by qRT-PCR after 3-day induction. Glyceraldehyde-3-phosphate dehydrogenase (GAPDH) was used as the normalisation control; (**c**) The protein levels of DSPP and DMP1 were measured by western blotting after 7-day induction. β-actin was used as the internal control; (**d**) ALP activity in cellular lysates was determined after 7-day odontogenic induction. (**e**) The formation of mineralized nodules was visualized by alizarin red staining at 7 days after induction. Scale bar: 100 μm. All data are presented as the mean ± SD (*n* = 3). * *p* < 0.05, ** *p* < 0.01, *** *p* < 0.001 vs. pcDNA3.1-NC or Control-NC group, # *p* < 0.05, ### *p* < 0.001 vs. OS-NC group.

To determine the functional role of RORα in the regulation of odontoblastic differentiation, we overexpressed RORα in rDPCs by transfecting them with pcDNA3.1-RORα. Cells transfected with pcDNA3.1-NC were used as the negative control. The efficiency of RORα overexpression was confirmed by qRT–PCR and western blotting, which demonstrated a marked upregulation of RORα at both the mRNA and protein levels in the RORα overexpression group when compared with the negative control (Figure 4a). 

Next, we assessed the effect of RORα overexpression on odontoblast-related genes. Twenty-four hours after transfection, rDPCs were cultured in maintained medium or OS medium for the specified time. qRT-PCR showed that after 3-day induction, the mRNA levels of DSPP, DMP1, and alkaline phosphatase (ALP) were significantly increased in the OS group (*p* < 0.001) (Figure 4b). When compared with the negative control group, the transcriptional levels of DSPP, DMP1, and ALP were significantly higher in the RORα overexpression group during odontogenic induction (*p* < 0.001), whereas only DSPP and ALP were upregulated in the maintained medium group (*p* < 0.01) (Figure 4b). Consistently, the protein levels of DSPP and DMP1 were increased after 7 days of odontogenic induction and were further upregulated by RORα overexpression (Figure 4c). Moreover, ALP activity was upregulated in the OS group on day 7, and ALP activity was much higher in the RORα overexpression group than in the negative control group (Figure 4d). To detect the effect of RORα overexpression on matrix mineralisation, alizarin red staining was performed. As demonstrated in Figure 4e, calcified nodules were only observed in the OS group after 7 days of odontogenic induction, and RORα overexpression led to a 1.5-fold increase in the formation of mineralized nodules. Thus, RORα played a positive role in the odontoblastic differentiation of DPCs.

### 2.5. Knockdown of RORα Inhibits Odontoblastic Differentiation in rDPCs

**Figure 5:**
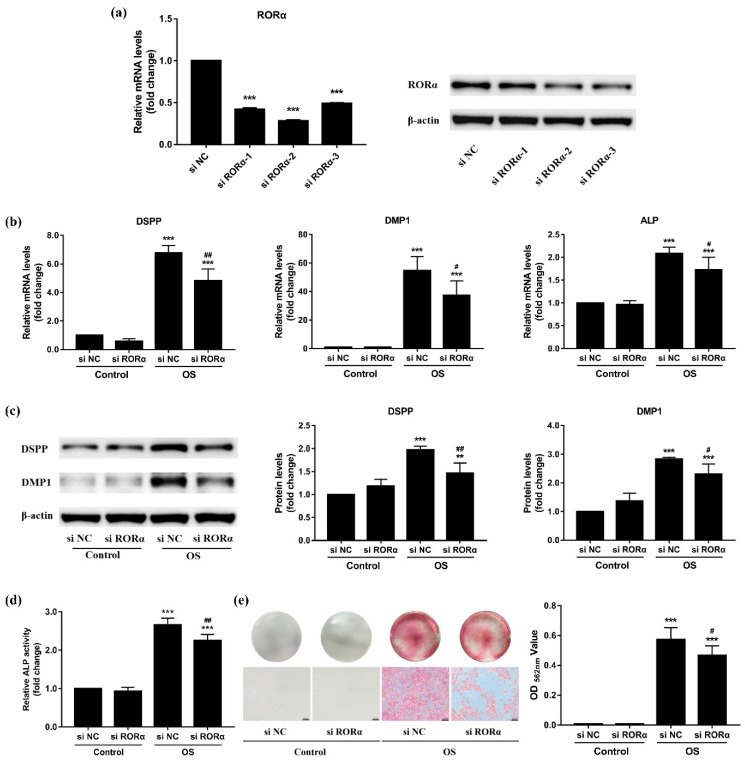
Effect of retinoid acid receptor-related orphan receptor α (RORα) knockdown on odontoblastic differentiation of rat dental papilla cells (rDPCs). rDPCs were transfected with small interfering (si) RORα or si negative control (NC) for 8 h and then cultured in control or odontogenic induction (OS) medium for 3 or 7 days. (**a**) The knockdown efficiency of RORα was examined by quantitative reverse transcription polymerase chain reaction (qRT-PCR) and western blotting; (**b**) The mRNA levels of dentin sialophosphoprotein (DSPP), dentin matrix protein 1 (DMP1), and alkaline phosphatase (ALP) were measured by qRT-PCR after 3-day induction. Glyceraldehyde-3-phosphate dehydrogenase (GAPDH) was used as the internal control; (**c**) The protein levels of DSPP and DMP1 were detected by western blotting after 7-day induction and normalised to β-actin levels; (**d**) ALP activity was analysed after 7 days of odontogenic induction. (**e**) The formation of mineralized nodules was assessed by alizarin red staining at day 7. Scale bar: 100 μm. All data are presented as the mean ± SD (*n* = 3). ** *p* < 0.01, *** *p* < 0.001 vs. si NC or Control-si NC group, # *p* < 0.05, ## *p* < 0.01 vs. OS-si NC group.

To confirm the effect of RORα on odontoblastic differentiation, we knocked down RORα using small interfering RNA (si RORα) in rDPCs. Cells in the negative control group were transfected with si NC. After transient transfection, both the mRNA and protein levels of RORα were efficiently downregulated when compared with those of the negative control group (Figure 5a). si RORα-2 yielded the highest knockdown efficiency (>70%); therefore, it was selected for subsequent experiments. 

As shown in Figure 5b, the mRNA levels of DSPP, DMP1, and ALP were upregulated after 3 days of odontogenic induction (*p* < 0.001), whereas in the OS group, their transcriptional levels were significantly attenuated in response to RORα knockdown (*p* < 0.05). Similarly, under odontogenic induction, the protein levels of DSPP and DMP1 were increased on day 7, but they were downregulated in the RORα knockdown group when compared with the negative control group (Figure 5c). ALP activity was notably upregulated in the OS group after 7-day induction; however, there was a notable decrease in ALP activity in the RORα knockdown group (Figure 5d). Moreover, as indicated by alizarin red staining, inhibition of RORα also greatly suppressed mineralized nodules formation after 7 days of odontogenic induction (Figure 5e). However, when rDPCs were cultured in maintained medium without odontogenic induction, the results did not differ between the si RORα and si NC groups (*p* > 0.05). Accordingly, RORα acted as a positive regulator of the odontoblastic differentiation of DPCs.

### 2.6. Melatonin Promotes Odontoblastic Differentiation of rDPCs in an RORα-Dependent Manner

**Figure 6:**
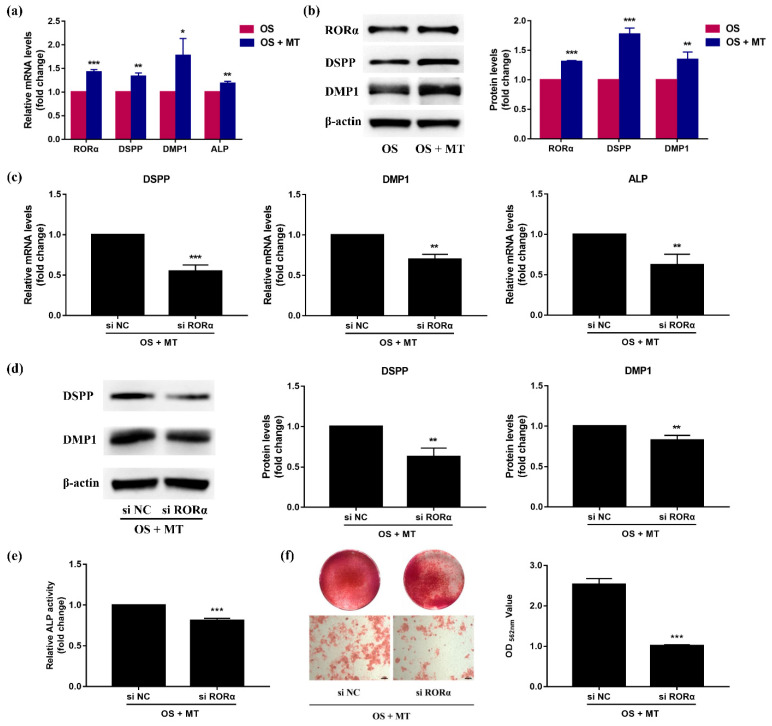
Effect of retinoid acid receptor-related orphan receptor α (RORα) on melatonin-induced odontoblastic differentiation of rat dental papilla cells (rDPCs). (**a**,**b**) rDPCs were stimulated with odontogenic induction (OS) medium in the presence or absence of 10−8 mol/L melatonin (MT) for 7 days. The mRNA levels of RORα, dentin sialophosphoprotein (DSPP), dentin matrix protein 1 (DMP1), and alkaline phosphatase (ALP) were quantified by quantitative reverse transcription polymerase chain reaction (qRT-PCR), and glyceraldehyde-3-phosphate dehydrogenase (GAPDH) was used as the normalised control (**a**); The protein levels of RORα, DSPP and DMP1 were measured by western blotting and normalised to β-actin levels (**b**). (**c**–**f**) rDPCs were transfected with small interfering (si) RORα or si negative control (NC) for 8 h and subsequently incubated in OS medium with 10−8 mol/L melatonin for 7 days. The mRNA levels of DSPP, DMP1 and ALP were examined by qRT-PCR, and GAPDH was used as the internal control (**c**); The protein levels of DSPP and DMP1 were determined by western blotting and normalised to β-actin (**d**); ALP activity in cellular lysates was assessed (**e**), and the formation of mineralized nodules was visualized by alizarin red staining (**f**). Scale bar: 100 μm. All data are presented as the mean ± SD (*n* = 3). * *p* < 0.05, ** *p* < 0.01, *** *p* < 0.001 vs. OS or si NC group.

Previous studies of our group have suggested that under odontogenic induction, melatonin promotes the odontoblastic differentiation of rDPCs and matrix mineralisation in a dose-dependent manner, with 10^−8^ mol/L melatonin displaying the optimal stimulative effect [31,32]. Thus, OS medium supplemented with 10^−8^ mol/L melatonin was used for our subsequent studies. To determine whether RORα mediates the pro-odontogenic effect of melatonin on rDPCs, we first examined whether melatonin induces RORα expression. We treated rDPCs with OS medium in the presence or absence of 10^−8^ mol/L melatonin for 7 days. As shown in Figure 6a,b, the mRNA and protein levels of RORα were significantly increased in the melatonin-treated group (*p* < 0.001), along with the apparent upregulation of DSPP, DMP1, and ALP (*p* < 0.05). 

Next, we performed another set of loss-of-function experiments in rDPCs using siRNA to knock down RORα expression, followed by incubation in OS medium with 10^−8^ mol/L melatonin for 7 days. Compared with the negative control group, the mRNA levels of DSPP, DMP1, and ALP were notably downregulated in the RORα knockdown group (Figure 6c). In addition, as demonstrated in Figure 6d, inhibition of RORα markedly attenuated the protein levels of DSPP and DMP1. Consistent with the reduced odontoblast-related markers, ALP activity analysis showed that silencing RORα significantly blunted the effects of melatonin on ALP activity (*p* < 0.001) (Figure 6e). Furthermore, after odontogenic induction with OS medium containing melatonin, alizarin red staining revealed calcium nodule deposition. However, RORα knockdown led to a 2.5-fold decrease in the mineralized nodule formation (Figure 6f). These data indicated that RORα knockdown attenuated melatonin-induced odontoblastic differentiation of DPCs, implying that melatonin promoted odontoblastic differentiation via the nuclear receptor RORα.

## 3. Discussion

In this study, we explored the role of the nuclear receptor RORα in odontoblastic differentiation. First, we demonstrated that endogenous RORα was expressed in rDPCs and was significantly upregulated during odontoblastic differentiation both in vitro and in vivo. Second, RORα overexpression was shown to promote the odontoblastic differentiation of rDPCs, whereas RORα knockdown inhibited it. Finally, our results revealed that RORα mediated the pro-odontogenic effect of melatonin. Collectively, our findings provide the first evidence that the nuclear receptor RORα is a novel positive regulator of the odontoblastic differentiation of DPCs and is a critical mediator in melatonin-induced odontoblastic differentiation.

Nuclear receptors, as DNA-binding transcription factors, can regulate the expression of specific target genes at the transcriptional level. These genes play fundamental roles in cell proliferation, differentiation, and embryonic development [52]. Multiple transcription factors have been shown to be pivotal in odontogenesis. For example, runt-related transcription factor 2 and osterix are considered vital transcriptional factors for odontoblastic/osteogenic differentiation [53,54], and other transcription factors, such as oestrogen receptor α, ATF6, and TRPS1, also affect the differentiation of stem cells into odontoblasts [55,56,57]. RORs, belonging to the nuclear receptor superfamily, comprise three specific isotypes: RORα, RORβ, and RORγ [33]. RORα is widely expressed in various tissues, including the brain, liver, heart, lungs, kidney, skin, adipose, and bone marrow [36,40,46,49]. RORβ displays a relatively restricted expression pattern, being limited to the brain, pineal gland, and retina [58,59]. In contrast, the expression of RORγ1 can be detected in a variety of tissues, such as the liver, kidney, adipose tissue, and skeletal muscle; however, the expression of RORγt (RORγ2) is extremely limited to the immune system [60,61]. Correspondingly, these subtypes exhibit different regulatory effects on cellular differentiation and tissue development. For example, RORα is implicated in the process of bone formation and regulates the development of the cerebellum [36,47]. In a previous study, RORβ-deficient mice exhibited retinal degeneration, indicating the role of RORβ in the development of the retina [59]. RORγ is essential for the formation of lymphoid tissues and regulates thymopoiesis [60,62]. However, to date, no studies have demonstrated the role of RORs in tooth development. In this study, primary rDPCs derived from the tooth germs of the first molars were selected because they are the precursors of odontoblasts and can differentiate into odontoblast-like cells under odontogenic induction in vitro [6]. Therefore, DPCs represent a reasonable model system for investigating the expression and function of RORs in the context of odontoblastic differentiation and tooth development. This study was the first to detect that RORα was highly expressed in rDPCs and that it was mainly located in the nuclei. In contrast, it was shown that the expression of RORβ was lower and RORγ was not expressed. This expression pattern indicated that RORα might be related to DPC differentiation.

Odontoblastic differentiation is a critical process of tooth development. During this process, DPCs are first induced to become preodontoblasts, followed by their elongation and polarisation to become immature odontoblasts, finally differentiating into mature odontoblasts. Differentiated odontoblasts, including immature and mature odontoblasts, synthesise and secrete extracellular matrix proteins to produce dentin [7]. The process of differentiation from DPCs to secretory odontoblasts involves many molecules at each stage. ALP is considered an early-stage marker of odontoblastic differentiation, which can facilitate mineral deposition [63,64]. DSPP and DMP1, members of the small integrin-binding ligand N-linked glycoprotein gene family, are highly expressed in mature odontoblasts and are essential for mineralisation of the extracellular matrix, as well as dentin formation [53,65,66]. Therefore, we selected DSPP, DMP1, and ALP as the specific markers for odontoblastic differentiation. In this study, after odontogenic induction for 3 or 7 days in vitro, the expression levels of DSPP, DMP1 and ALP were found to be remarkably upregulated, indicating that rDPCs were undergoing odontoblastic differentiation; these results were consistent with those of our previous study [13]. Furthermore, we observed that the expression of RORα significantly increased at both the mRNA and protein levels during this process. Through immunochemical analysis, we found that RORα protein was gradually upregulated along with the progression of odontoblastic differentiation in vivo and was strongly expressed in differentiated odontoblasts. These observations were in agreement with those of previous studies, which showed that RORα is upregulated in human bone marrow mesenchymal stem cells during osteogenic differentiation [46,47]. More importantly, these observations suggested that RORα is positively correlated with odontoblastic differentiation and tooth development.

Mounting evidence has shown the key role of RORα in both cellular differentiation and bone metabolism. Previous functional studies have demonstrated that RORα overexpression can improve the expression of osteogenic markers, including ALP, bone sialoprotein (BSP), DMP1, osteocalcin, and collagen type I, and promote the formation of mineralized nodules during osteogenesis, whereas RORα suppression inhibits these responses [44,46,67]. Meanwhile, it has also been reported that RORα acts as a negative regulator of adipocyte differentiation, which was indicated by the reduced expression of adipogenic genes and decreased lipid accumulation [42,68]. Notably, RORα, as a transcription factor, could directly bind to ROREs in the promoter regions of target genes to regulate gene expression [34]. For instance, some studies revealed that RORα activates the promoter activity of BSP and bone morphogenetic protein 2, which are vital for osteogenic differentiation [47,67], whereas it suppresses the promoter activity of perilipin by competing with peroxisome proliferator-activated receptor gamma (PPARγ), thus inhibiting PPARγ-dependent adipogenesis [68]. It has also been demonstrated that there is a delicate balance among transcription factors to determine the lineage differentiation of stem cells, which is consistent with the different regulatory effects of RORα on osteogenesis and adipogenesis [69]. In addition, RORα can inhibit bone resorption by suppressing TNFα-induced inflammatory responses [44,45]. Furthermore, in vivo experiments showed that RORα-knockout mice exhibit marked abnormalities in bone tissue, which are manifested by thin long bones and reduced mineral content [47]. Although these findings strongly indicate that RORα positively regulates osteogenic differentiation and bone development, its specific functions in odontoblastic differentiation and tooth development have not been investigated. Odontogenesis is similar to osteogenesis; for example, odontoblasts and osteoblasts share many similar properties, including the expression of relevant genes and the production of calcified nodules in vitro. In this study, we observed the increased expression of RORα in differentiated odontoblasts; thus, we hypothesised that RORα acts as a positive regulator of odontoblastic differentiation. To test our hypothesis, we performed a series of gain- and loss-of-function studies. We observed that overexpression of RORα could stimulate the expression of DSPP and DMP1 at both the mRNA and protein levels and enhance the mRNA level of ALP as well as ALP activity. Additionally, overexpression of RORα could facilitate the formation of mineralized nodules after odontogenic induction. In contrast, knockdown of RORα attenuated the upregulation of those genes in the presence of OS medium. ALP activity and mineralisation was affected in a similar manner. Taken together, this is the first report showing that RORα promotes the odontoblastic differentiation of rDPCs.

Initially, RORα was considered an orphan nuclear receptor without endogenous ligands; however, after a series of studies, melatonin is now regarded as a moderate-affinity ligand for RORα [70,71]. Although whether RORα is a true nuclear receptor of melatonin remains debatable [72], RORα mediates many important physiological and pharmacological effects of melatonin, including circadian rhythm, oxidative stress, and immune response [48,50,73]. Melatonin is a multifunctional molecule involved in various biological processes [14,15,16,17,18,74]. Recently, melatonin has become an important research area, with the primary focus on its potential in regenerative medicine [22]. Numerous studies have shown that melatonin is implicated in the development and regeneration of various tissues, including the bone, muscle, skin, hair, liver, kidney, bladder, and nervous system [22,23,24,75,76]. However, research on the effects of melatonin on tooth development and regeneration is limited. For example, it has been reported that melatonin has effects on the biological and immunomodulatory properties of human dental pulp stem cells, suggesting the potential clinical use of melatonin for attenuating inflammation and promoting dental tissue regeneration in oral diseases [77]. In addition, we previously demonstrated that physiological concentrations of melatonin, especially at 10^−8^ mol/L, promotes the differentiation of DPCs into odontoblasts, although, the precise mechanism remains unknown [31,32,78]. Melatonin mainly acts through membrane receptors (MT1 and MT2), nuclear receptors (RORα), cytosolic binding sites and mitochondria [19,20,21]. However, we previously found that luzindole, an MT1/MT2 receptor antagonist, cannot inhibit melatonin-induced odontoblastic differentiation, excluding the possibility of a membrane receptor-dependent mechanism [31]. Next, we focused on the potential role of RORα. We observed that 10^−8^ mol/L melatonin enhanced the expression of DSPP, DMP1, and ALP, which is consistent with the findings of previous studies [31,32]. Notably, this process was accompanied by the upregulation of RORα. To further verify that melatonin exerts its pro-odontogenic effect via the nuclear receptor RORα, we knocked down RORα in rDPCs cultured in OS medium containing melatonin. Consequently, suppression of RORα decreased the expression of DSPP, DMP1, and ALP and blunted the melatonin-induced ALP activity and matrix mineralisation. Together, these data indicate that melatonin promotes the odontoblastic differentiation of DPCs in an RORα-dependent manner. Meanwhile, our laboratory noticed that the process of melatonin-induced differentiation of DPCs was accompanied by changes in mitochondrial function and biogenesis [31,32]. Given that RORα can affect mitochondrial function, fission and biogenesis, and can regulate the transcription of mitochondria-related genes via direct binding to their ROREs [79,80,81], further studies are still required to clarify whether RORα mediates the pro-odontogenic effect of melatonin through mitochondria.

In conclusion, the present study provides the first evidence that the nuclear receptor RORα is a positive regulator of odontoblastic differentiation and an important mediator of melatonin-induced DPC differentiation into odontoblasts. These findings not only elucidate the downstream mechanism of the pro-odontogenic effect of melatonin but also provide a potential target for dental tissue regeneration.

## 4. Materials and Methods

### 4.1. Cell Isolation and Culture

All animal experiments were approved by the Ethics Committee of Zhongshan School of Medicine, Sun Yat-sen University, China (No.2017-218). The dental papilla was gently isolated from the first molar of 1-day postnatal Sprague-Dawley rats (purchased from Sun Yat-sen University, Guangzhou, China) under a stereomicroscope (Stemi2000, Zeiss, Jena, Germany) and minced into small pieces (approximately 1 mm^3^ in size). The tissue pieces were then seeded on 10-cm culture dishes and maintained in α-minimal essential medium (α-MEM; Gibco, Grand Island, NY, USA) containing 20% fetal bovine serum (FBS; Gibco, Grand Island, NY, USA), 100 U/mL penicillin, and 100 μg/mL streptomycin (Gibco, Grand Island, NY, USA) at 37 °C in 5% CO_2_ humidified air. Upon reaching 80–90% confluence, cells were passaged using TrypLE (Gibco, Grand Island, NY, USA) and purified by a distinct digestion method. Cells within 3–4 passages were used for subsequent experiments.

For odontoblastic differentiation, rDPCs were cultured in an osteogenic/odontogenic induction medium (OS; α-MEM supplemented with 10% FBS, 1% antibiotics, 0.1 mM dexamethasone, 0.2 mM ascorbic acid, and 10 mM β-glycerophosphate; Sigma-Aldrich, St. Louis, MO, USA). The induction medium was changed every 2 days. The cells were harvested for qRT-PCR, western blotting, ALP activity assays, and alizarin red staining at different time points.

### 4.2. Cell Transfection

To knockdown RORα expression, DPCs were seeded in 12-well plates at a density of 7.5 × 10^4^ cells per well. Upon reaching 30–50% confluence, small interfering RNA (siRNA) targeting rat RORα (si RORα, GenePharma, Suzhou, China) or negative control siRNA (si NC, GenePharma, Suzhou, China) were transfected into DPCs at a final concentration of 100 nM with RNAFit transfection reagent (Hanbio, Shanghai, China) for 8 h according to the manufacturer’s protocol. To overexpress RORα expression, DPCs were seeded in 12-well plates at a density of 1.5 × 10^5^ cells per well. The next day, when cells reached 60–80% confluence, they were transfected with 1 μg expression vector encoding rat RORα (pcDNA3.1-RORα, GenePharma, Suzhou, China) or 1 μg empty vector (pcDNA3.1-NC, GenePharma, Suzhou, China) per well using NeofectTM DNA transfection reagent (Neofect Biotech, Beijing, China) according to the manufacturer’s instructions. Twenty-four hours after transfection, the serum-free medium was replaced with maintained medium or OS medium.

### 4.3. Immunofluorescence Staining

DPCs seeded on 35 mm glass bottom dishes were fixed with 100% cold methanol (−20 °C) for 5 min at room temperature and were blocked in 5% bovine serum albumin (BSA) for 1 h. Subsequently, DPCs were incubated with anti-RORα primary antibody (1:200, Abcam, Cambridge, UK) at 4 °C overnight, followed by incubation with Dylight 488-conjugated secondary antibody (1:200, EarthOx, CA, USA) for 1 h at room temperature in the dark. The nuclei were stained using 4-6-diamidino-2-phenylindole (DAPI; Roche, Basel, Switzerland) for 5 min in the dark. The cells were then visualised under a laser scanning confocal microscope (LSM 780; Zeiss, Jena, Germany).

### 4.4. Immunohistochemistry

The mandibles were dissected from 1-day postnatal Sprague-Dawley rats (purchased from Sun Yat-sen University, Guangzhou, China), fixed in 10% buffered paraformaldehyde for 48 h, and decalcified with 10% ethylenediaminetetraacetic acid (pH 7.4) for 5 days. After dehydration and paraffin embedding, the tissues were cut into 5-μm sagittal sections. Tissue sections were deparaffinized, rehydrated, and heat-retrieved in 0.01 mol/L citrate buffer (pH 6.0) for 15 min at 100 °C followed by cooling at room temperature. Subsequently, they were incubated in 3% hydrogen peroxide for 15 min at room temperature, blocked with 5% BSA for 1 h, and then incubated with anti-RORα primary antibody (1:100, Abcam, Cambridge, UK) at 4 °C overnight. After washing with PBS, the specimens were incubated in Polymer Helper (Bioss, Beijing, China) and horseradish peroxidase (HRP)- anti-rabbit IgG (Bioss, Beijing, China) at 37 °C for 20 min and then visualised using diaminobenzidine in the dark and counterstained with haematoxylin. Finally, the slides were dehydrated, transparentised with dimethylbenzene, and sealed using a mounting medium. The slides were observed using a digital pathology slide scanner (Aperio AT2; Leica Biosystems, Wetzlar, Germany).

### 4.5. RNA Isolation, Quantitative Real-Time Polymerase Chain Reaction and Agarose Gel Electrophoresis

Total RNA was isolated from DPCs using an RNA-Quick purification kit (YISHAN Biotechnology, Shanghai, China) according to the manufacturer’s instructions. For qRT-PCR, first-strand cDNA was synthesised using PrimeScriptTM RT Master Mix Kit (TaKaRa, Dalian, Japan) and then amplified with SYBR Green I Master Mix (Roche, Basel, Switzerland) in the LightCycler 480 Real-Time PCR System (Roche, Basel, Switzerland). The relative mRNA expression of target genes was normalised to that of glyceraldehyde-3-phosphate dehydrogenase. For the agarose gel electrophoresis assay, amplified RT-PCR products were synthesised using the HiScript II One Step RT-PCR Kit (Vazyme, Nanjing, China) according to the manufacturer’s protocol, then separated on a 2% agarose gel and visualised with a gel imaging system (BioDoc-It2 Imager; Analytik Jena US LLC, Jena, Germany). The specific primers used in this study are listed in Table 1.

### 4.6. Western Blotting

Total protein was extracted from the cells using a radio-immunoprecipitation assay lysis buffer (RIPA) supplemented with protease and phosphatase inhibitors (Cwbio, Beijing, China) and measured with a bicinchoninic acid (BCA) protein assay kit (Cwbio, Beijing, China). Equal amount of protein lysates (20–30 μg/lane) were separated by 4–12% sodium dodecyl sulfate polyacrylamide gel electrophoresis and transferred onto polyvinylidene fluoride membranes (Millipore, Billerica, MA, USA). Then, the membranes were blocked with 5% non-fat milk for 2 h at room temperature. Subsequently, they were incubated overnight at 4 °C with the following primary antibodies: polyclonal rabbit anti-RORα antibody (1:2000, PA5-23268, Thermo Scientific, MA, USA), monoclonal mouse anti-DSPP antibody (1:500, sc-73632, Santa Cruz, CA, USA), polyclonal rabbit anti-DMP1 antibody (1:1000, NBP 1-45525, Novus Biologicals, Littleton, CO, USA), and monoclonal mouse anti-β-actin (1:1000, AF0003, Beyotime, Shanghai, China). After washing with tris-buffered saline and tween 20 (TBST), the membranes were incubated with the corresponding HRP-conjugated secondary antibodies (1:2000, A0216, A0208, Beyotime, Shanghai, China) at room temperature for 1 h. The immunoreactive bands were detected using chemiluminescence detection reagents (Millipore, Temecula, MA, USA) and visualised using an ImageQuant LAS 4000 mini system (GE Healthcare Life Sciences, Chicago, IL, USA). The intensities of the bands were quantified using ImageJ 1.36b (NIH, Bethesda, MD, USA).

### 4.7. Alkaline Phosphatase Activity

DPCs were cultured in OS medium for 7 days. ALP activity in cellular lysates was measured using an ALP activity detection kit (Beyotime, Shanghai, China) according to the manufacturer’s instructions. The protein concentration was quantified using a BCA protein assay kit (Cwbio, Beijing, China). One unit of ALP activity was defined as the amount that liberated 1 mol p-nitrophenol per mg protein.

### 4.8. Alizarin Red Staining

DPCs were seeded into 12-well plates and cultured in OS medium for 7 days. After fixation in 4% paraformaldehyde for 30 min, DPCs were stained with 1% alizarin red staining solution (ARS; Cyagen, Suzhou, China) for 5 min at room temperature and were then rinsed with distilled water. The matrix calcium deposition was scanned using an inverted phase-contrast microscope (Axio 40; Zeiss, Jena, Germany). To quantify the mineralized nodules, the stained cells were incubated in 0.1M hexadecylpyridinium chloride monohydrate (Sigma-Aldrich, St. Louis, MO, USA) for 30 min. The absorbance of the supernatant was then measured at a wavelength of 562 nm.

### 4.9. Statistical Analysis

All data are presented as the mean ± standard deviation of triplicate independent experiments. Two-group comparisons were assessed using Student’s two-tailed *t*-test. Multiple group comparisons were conducted by one-way analysis of variance followed by Fisher’s least significant difference post hoc test. Statistical analysis was performed using SPSS 22.0 software (SPSS, Inc., Chicago, IL, USA) and *p* < 0.05 was considered statistically significant.

## Figures and Tables

**Table 1:** Specific primers for polymerase chain reaction (PCR).

Gene	Forward Primer (5′-3′)	Reverse Primer (5′-3′)
RORα	CTACCAGAACAAGCAGAGA	CGAACTCCACCACATACT
RORβ	ATCCGCTAACAGGCACAGATG	AGGAAAGAAAGAAAGGCGGCA
RORγ	CTGGCTGCAAAGAAGACCCA	CCCGTAGTGGATGCCAGATG
DSPP	ACAGCGACAGCGACGATTC	CCTCCTACGGCTATCGACTC
DMP1	CTGGTATCAGGTCGGAAGAATC	CTCTCATTAGACTCGCTGTCAC
ALP	GGAAGGAGGCAGGATTGA	TCAGCAGTAACCACAGTCA
GAPDH	TATGACTCTACCCACGGCAAGT	ATACTCAGCACCAGCATCACC

## Data Availability

The data presented in this study are available on request from the corresponding author.

## Sample Availability

Samples of the compounds are available from the authors.
